# Promoting and inhibiting factors for the implementation of evidence-based non-invasive treatment programs for patients with knee- and/or hip-osteoarthritis: a rapid review

**DOI:** 10.1186/s12913-026-14219-5

**Published:** 2026-03-04

**Authors:** Jennifer Bosompem, Peter Rasche, Nino Chikhradze, Barbara Jömann, Horst Christian Vollmar, Ina Carola Otte, Theresa Sophie Busse

**Affiliations:** 1 Institute of General Practice and Family Medicine (AM RUB), Ruhr University Bochum, Bochum, Germany; 2 Institute for Diversity Medicine, Ruhr University Bochum, Bochum, Germany; 3 Digital Health, Witten/Herdecke University, Witten, Germany; 4 Department of Healthcare, University of Applied Sciences – Hochschule Niederrhein, Krefeld, Germany

**Keywords:** Implementation science, Rapid review, Osteoarthritis, Osteoarthrosis, Osteoarthritis, Hip Joint, Knee Joint, Hip, Osteoarthritis, Knee

## Abstract

**Background:**

Osteoarthritis of the knee and/or hip joint are common conditions among older adults. Although multimodal therapy combining patient education, exercises and weight loss has proven effective, only a few countries offer implemented treatment programs. While some Scandinavian countries have achieved nationwide implementation, similar initiatives remain limited to research settings elsewhere. To date, limited evidence exists on factors that facilitate or hinder the successful implementation of these programs. This study therefore aims to synthesize scientific evidence on the implementation of evidence-based, non-invasive treatment programs for osteoarthritis of the knee and/or hip joint.

**Question:**

What is the current state of international research on the implementation of evidence-based, non-invasive treatment programs for osteoarthritis of the knee and/or hip joint, and, which promoting and inhibiting factors regarding implementation have been reported?

**Design:**

Rapid review.

**Literature search:**

The search term was derived using the PICo-scheme. Searches were conducted in PubMed, PEDro, Embase and Cochrane in September 2022. Reference lists of included titles were screened manually for relevant publications.

**Study selection:**

Eligible studies reported on the implementation of non-invasive treatment programs for patients with osteoarthritis of the knee and/or hip joint including education and training and were published between 2008 and 2022 with full-text-availability. Studies focusing on in-patient services, competitive sports or languages other than English or German were excluded. Screening was supported by Rayyan and ASReview LAB.

**Data synthesis:**

A qualitative content analysis according to Kuckartz using MAXQDA was performed.

**Results:**

The search identified 5,948 records. After screening, 40 studies were assessed in full text, of which 11 studies were included for data extraction. Four key dimensions relevant to implementation were identified: The scientific, political, organizational, and financial dimension. Promoting factors included scientific monitoring during implementation, central program support, cooperation between implementation teams, gradual implementation, free patient access to lower participation barriers, broad availability and efficient referral. Inhibiting factors comprised restrictive health policy frameworks, missing public funding for implementation costs and service provision, insufficient staff, incompatibility with existing care structures, insufficient coordination and management and limited authority of healthcare professionals in regional contexts.

**Conclusion:**

The rapid review highlights the multifactorial nature of the factors influencing the implementation of treatment programs and underscores the need to link practice and research.

**Supplementary Information:**

The online version contains supplementary material available at 10.1186/s12913-026-14219-5.

## Background

Osteoarthritis (OA) of knee and hip joints is a frequent problem for the elderly; about 40% of the over 65-year-old suffer from pain of the knee and/or hip joints [1]. A multimodal therapy concept consisting of patient education, exercise program and weight loss is favored in international literature and in various guidelines [2, 3].

Statistics emphasize that many patients are affected: In German-speaking countries, a prevalence of 23.8% for knee arthrosis (gonarthrosis) and 15–20% for hip arthrosis (coxarthrosis) is reported in people over the age of 60 [4, 5]. Data from 2020 show that musculoskeletal diseases rank fifth in health care costs in Germany after diseases of the circulatory system, mental and behavioral disorders, digestive system diseases and neoplasms [6]. However, these diseases are becoming increasingly relevant for healthcare systems and patients around the whole world. Therefore, some countries already provide treatment options for OA apart from surgical procedures. Over the past ten years, there have been strong efforts in some countries to establish research findings on evidence-based treatment of mild to moderate OA in practice: Initiatives such as “Better Management of patients with OsteoArthritis” (BOA) [7] from Sweden or “Good Life with osteoArthritis in Denmark” (GLA:D^®^)^8^ have similar goals. In the accompanying studies to GLA:D^®^, a significant reduction in the risk of being unable to work due to OA of the knee and/or hip joint was measured [8]. GLA:D^®^ adapted the Swedish program for the Danish region and added a systematic, supervised exercise program specifically targeted at knee and hip pain (NEuroMuscular EXercise, NEMEX) [9]. The effect of this training program was assessed by specific functional tests, which were added to pain and quality-of-life scales during the evaluation. Meanwhile, the GLA:D^®^ program is also applied in other countries such as Switzerland, Canada, Australia and China [10]. Analysis of data from the GLA:D^®^ program from 2013 to 2015 in Denmark showed improvement in pain intensity and physical resilience. Further, physical activity improved within the first three months, use of pain medication decreased, and work disability was reduced [8]. While programs in the past have been tedious and expensive, newer programs are safe, effective, affordable, and feasible [11]. However, the supply of such evidence-based, non-invasive programs for the treatment of OA of the knee and/or hip joint in standard care is not widespread, despite OA being a global challenge [12]. Although research projects for the development of such programs exist, it is remarkable that they are not permanently implemented in standard care. What might contribute to this circumstance is that OA care programs are complex interventions, as any primary care intervention in healthcare [13]. Lau et al. developed a four-level framework in a review of reviews on the evidence to practice gap for complex interventions in healthcare and named the external context, the organization, the professionals and the intervention as relevant levels [14]. They recommend considering contextual factors when planning for successful implementation [14]. Swaithes et al. attribute the lack of implementation to a discrepancy between the research reality and the real world, which prevents those research programs from being realized [15]. Hurley et al. assume that the implementation does require extensive resources as well as effort and commitment [11].

A content analysis conducted by Datta & Petticrew examined the challenges involved in the design, implementation, and evaluation of complex interventions (including an OA program) [16]. They found that the information in studies is often insufficient in terms of outcomes, context, and intervention [16]. In addition, the authors reported that standardization and treatment fidelity were often challenging in the studies – both for providers and patients [16]. The authors also identified responsibilities, structural, and logistical challenges as critical issues [16]. This allows for an assessment of the possible results of this study, which takes an in-depth look at the field of OA programs [16].

For our rapid review, the word implementation describes the integration into standard care, which enables the utilization of the program by patients outside of research projects. The programs can be financed by public or private health insurance funds as well as out-of-pocket services.

To improve patient care, it is essential that healthcare programs are implemented regionwide within the frame of existing healthcare systems instead of temporary trials in studies. To this end, it is crucial to investigate existing barriers and how implementation can be accelerated. This is of particular interest regarding the adaptation of an existing treatment program from one country into another and with regard to the implementation of a new program.

To date, no reviews exist on the challenges and opportunities of implementing OA treatment programs as well-established services in standard care for entire regions or even countries. Therefore, indications can only be derived from general implementation research in the healthcare sector. In general implementation research in healthcare, it has already been acknowledged that implementation strategies must be tailored to the target population, the setting, and the goals for improvement. These findings can potentially be applied to the field of research on the implementation of evidence-based, non-invasive treatment programs for OA. However, it is essential to understand the specific challenges in the field of OA care to ensure individualized solutions, to improve care, and enable implementation. This is also consistent with the call for the redesign of implementation research to enhance scientific progress for patients and populations [17].

For these reasons, we conducted the present review to specify general findings from implementation research for the case of OA treatment programs. We achieve this specification by highlighting implementation barriers that can be considered in future implementation attempts. The study therefore aims to collect scientific evidence on implementation of evidence-based, non-invasive treatment programs for patients with OA of the knee and/or hip joint. The study results served as the basis for an interviewstudy with experts, in which the specific challenges identified in this study were further addressed (currently under review). The interview study was designed to generate in depth and international evidence on how to approach these challenges in more detail and to enable a comparison of the specific features of digital, analog, and blended care treatment programs and regional, national, and international hurdles. Therefore, the review was guided by the questions:

What is the current state of international research on the implementation of evidence-based, non-invasive treatment programs for patients with OA of the knee and/or hip joint?

What promoting and inhibiting factors have been reported regarding the implementation of the programs?

## Method

We chose the rapid review approach as it aligns with the systematic synthesis of knowledge commonly found in systematic reviews while the process is accelerated for quicker results [18]. The rapid review was registered prospectively at PROSPERO-International prospective register of systematic reviews from the National Institute for Health Research (CRD42022353957). No additional protocol was prepared. The review, including the development of the search strategy, the initial search, the screening as well as the qualitative analysis of the included literature, was conducted from September 2022 to August 2023 by two researchers (JB, TSB) and follows the recommendations on how to conduct a rapid review [19]. The reporting of the review adheres to the PRISMA Statement, given that the forthcoming PRISMA-RR Statement for rapid reviews is still under development [20].

### Deviations from the PROSPERO registration

Differing from the method described in the PROSPERO registration, the data extraction was based on a qualitative content analysis due to the heterogeneity of the included articles. A systematic review using the mixed-methods appraisal tool, as initially intended in the PROSPERO registration, was not carried out due to the small number of articles.

### Search strategy

The search was carried out in the databases PubMed, PEDro, Embase, and Cochrane. In addition, the reference lists of the included titles were searched by hand to identify relevant publications.

**Fig. 1:**
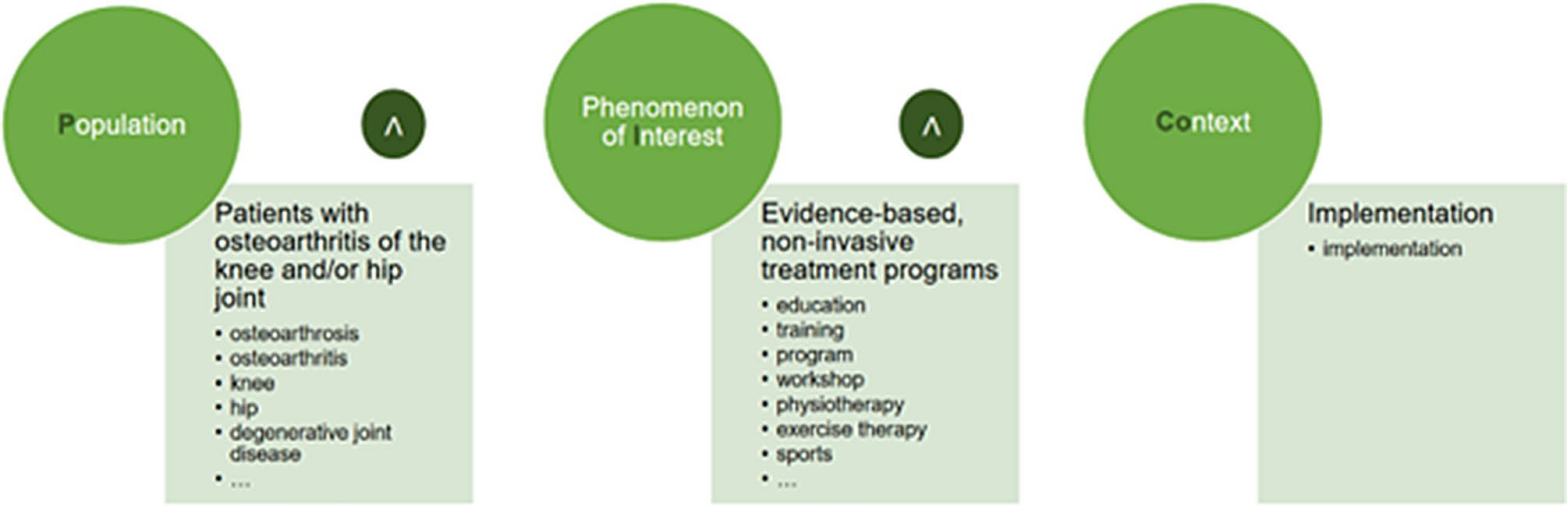
Search term based on PICo

The search term was derived by using the PICo scheme which can be seen in Fig. 1 - Search term based on PICo [21]. This scheme was selected as the aim of the review was not to analyze intervention effects or to compare interventions, but to examine the context of their implementation. By defining the **P**opulation (patients with OA of the knee and/or hip joint), Phenomenon of **I**nterest (evidence-based, non-invasive treatment programs) and **Co**ntext (implementation) the relevant keywords could be identified according to the research question.

The search term was extended by identifying additional synonyms using COREMINE medical, the Medical Subject Headings terms (MeSH)-browser and thesaurus and including database specific keywords and alternative spellings. Subsequently, the search term was discussed with two co-authors. Additionally, an expert from the library of the Ruhr University Bochum advised the authors on the technical adaptation of the search operators for the different databases. The final search was performed by two reviewers (JB, TSB) on 2022/09/28 using the search strings published on searchRxiv ( https://www.cabidigitallibrary.org/action/doSearch?AllField=Theresa+Busse&SeriesKey=searchrxiv

### Eligibility criteria

The eligibility of the articles was evaluated according to the eligibility criteria described in the PROSPERO-registration. Inclusion and exclusion criteria are presented in Table 1. Studies were eligible for inclusion if they described the implementation of a non-invasive treatment program for patients with OA of the knee and/or hip joint. In the context of this work, implementation was defined as the integration of the program into standard care. In order to consider existing guideline recommendations for the treatment of OA of the knee and/or hip joint [2, 3], only programs with an education and training component were included in the review. The temporal limitation of the publication period is aligned with the introduction of the programs Better Management for Osteoarthritis (BOA) in 2008 and GLA:D^®^ Denmark in 2013 as two of the first examples for a successful implementation of guideline-based treatment programs for OA.

**Table 1:** Inclusion and exclusion criteria

Languages	Inclusion criteria	Exclusion criteria
	English, German	Languages other than German or English
Publication period	2008–2022	Before 2008
Format	• Full text available	• Only abstract available • Full text not available
Study design	• Empirical studies on the efficacy of non-invasive treatments of patients with osteoarthritis • Randomized controlled trials • Empirical studies • Systematic Reviews • Study protocols • Gray Literature • Conference papers • Books • Academic theses (dissertation etc.)	• Bachelor thesis, master thesis, or similar works
Setting	International, out-patient services	In-patient services
Target group of the programme	Patients with • Osteoarthritis of the knee- and/or hip joint • Any severity of the disease • Any age	Focus on competitive sports
Design of the programme	At least education and training as components	

### Selection process

The screening process was conducted with technical support from the programs Rayyan (http://rayyan.qcri.org; Rayyan, Cambridge, USA) and ASReview LAB (https://asreview.nl; ASReview LAB developers, Utrecht, Netherlands). Rayyan promotes a faster screening process through its semi-automation features that support the title-abstract-screening and collaborative work on a review [22]. The active learning model of ASReview LAB ranks titles and abstracts according to the probability of their inclusion [23]. The model continuously updates the ranking based on an initial training dataset and the screening decisions of the reviewer [23]. According to a predefined stopping rule, the screening process is terminated as soon as only irrelevant publications are expected in the ranking [23]. The training data set is generated by labelling at least one included and one excluded publication as such [23, 24]. For the screening process, the oracle mode and the naïve Bayes classifier were set.

**Fig. 2:**
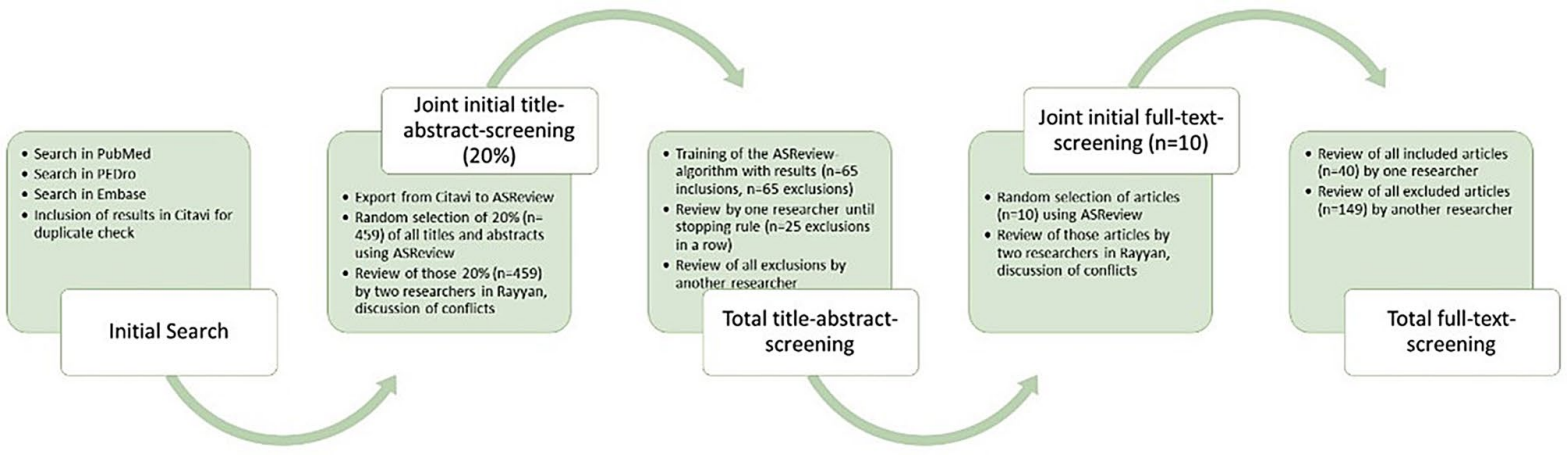
Selection process in the rapid review

The selection process was performed as illustrated in Fig. 2 – Selection process in the rapid review.

After carrying out the search in the four databases, duplicates were removed with the reference management program Citavi (https://www.citavi.com; Lumivero, Denver, USA). 20% of the remaining studies (*n* = 459) were then randomly selected and screened independently by two reviewers (JB, TSB) using Rayyan. This initial screening is intended to verify the inclusion and exclusion criteria and to ensure interrater reliability. Subsequently, one reviewer (JB) performed the total title-abstract-screening using ASReview LAB as described above. To generate the training data set, all 65 included titles from the 20% screening were marked as inclusions in ASReview LAB. In turn, 65 of the 394 exclusions from the 20% screening were randomly entered into ASReview LAB to ensure a balanced training data set [25]. The training data set was compiled from the results of the 20% screening to achieve a high quality of the data set. After the initial training of the program, one reviewer (JB) continued the remaining title-abstract-screening with ASReview LAB. The screening process was terminated after 25 consecutive exclusions as further inclusions were then considered unlikely [25]. All abstracts excluded by JB were cross-checked by a second reviewer (TSB), and conflicts were solved. To prepare the full-text-screening, 10 randomly selected publications were screened independently by the two reviewers, and conflicts were resolved. The remaining full texts were screened by TSB using Rayyan, while JB cross-checked all excluded full texts. Conflicts regarding the screening decision were again discussed afterwards between JB and TSB. No third person was needed to clarify the inclusion or exclusion.

### Data extraction

The analysis was performed using MAXQDA (https://www.maxqda.com; VERBI GmbH, Berlin, Germany) by TSB and JB.

In the first step of the analysis, JB and TSB each created a separate file with all included studies. Based on the steps of Kuckartz qualitative content analysis [26] they inductively derived the categories independently. In doing so, they developed both “factual categories” and “content and thematic categories” according to Kuckartz [26]. Factual categories refer to specific facts in the included studies [26]. Factual categories can, for example, be explicit about numerical information about program costs or named professions. Content and thematic categories structure the content by indicating topics [26]. These are, for example, categories that relate to the description of the design of a program’s expansion.

In the next step, the categories were all discussed in regular meetings by JB and TSB. Together, the category system was further refined and developed as a joint effort through continuous consensus building. JB and TSB reviewed all findings to ensure the information was coded appropriately. Conflicts were discussed and resolved in consultation with a third person (HCV). This resulted in one category system, presented in the results section.

In a third step, data on authors, year of publication, country, aim of the study, methods, sample and participants as well as the specific OA program mentioned were extracted. This was extracted by JB and TSB. The metadata table was checked twice by a student assistant.

## Results

### Flow of studies through the rapid review

**Fig. 3:**
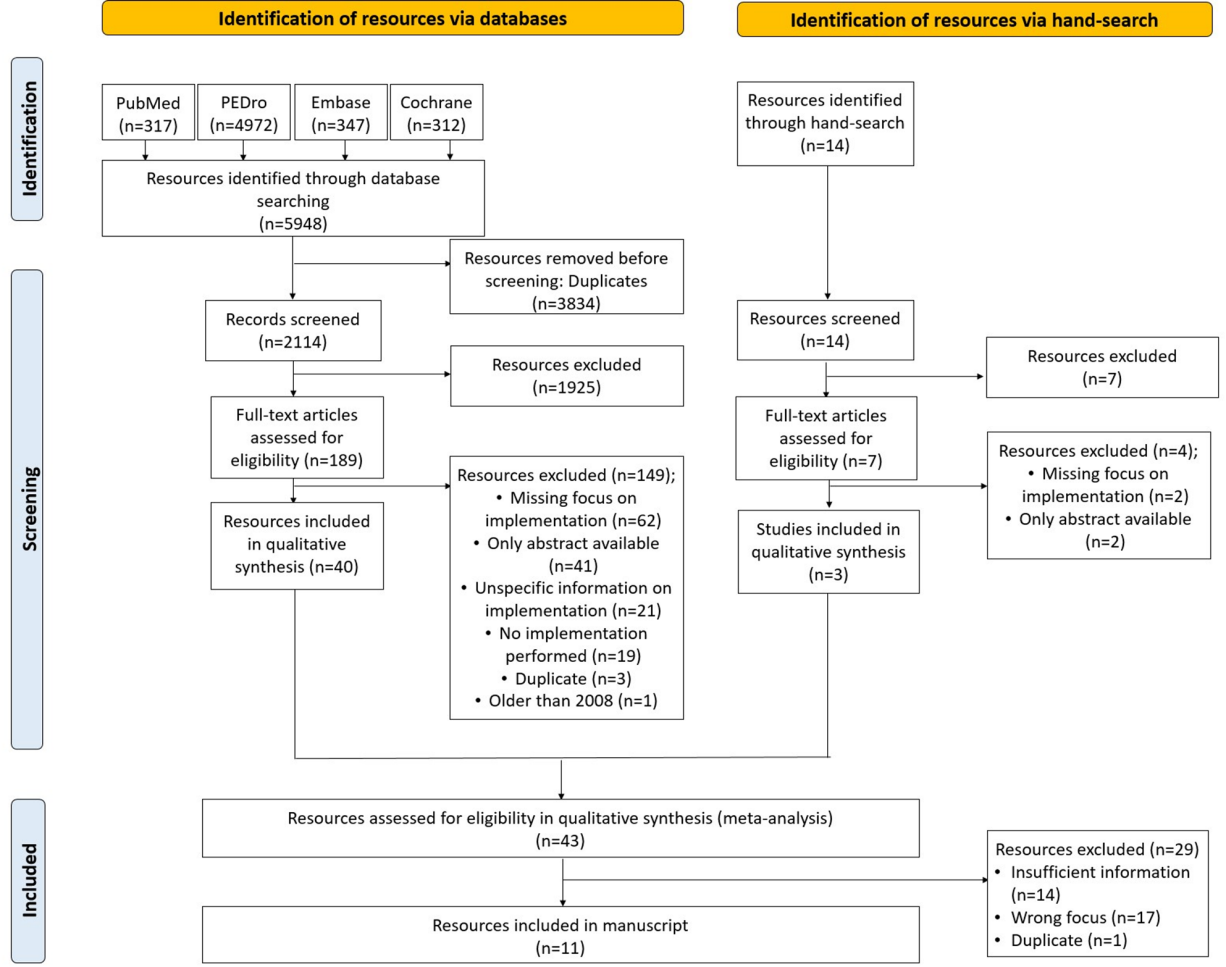
Flow chart

The initial search resulted in a total of 5,948 records of which 2,114 records remained for title-abstract-screening after removing the duplicates. After the title-abstract-screening, 189 records were eligible for the full-text-screening. The screening procedure was previously described in subsection *Selection Process*. The full-text-screening led to 40 remaining studies eligible for the final assessment of inclusion. An additional hand-search identified 14 further records of which 3 remained for the final assessment after title-abstract-screening and full-text-screening. Therefore, a total of 43 studies passed the final assessment of inclusion of which 11 were finally included for data extraction [27–37]. The study selection is described in Fig. 3 – Flowchart.

The included studies were reviewed with a particular focus on their relevance to the research question. This served to ensure the inclusion of all relevant information on the research question. In addition, a hand- search was carried out to trace the sources cited in the included articles. Extensive information can be found on searchRxiv.

### Study characteristics

The 11 included articles are all peer-reviewed scientific publications on recommendations or guidelines as well as articles on process evaluation, systematic reviews, narrative reviews, studies on effectiveness as well as qualitative studies:

Allen et al. (2016) as well as Bowden et al. (2020) compare and discuss OA programs regarding guidelines [27, 29]. Briggs et al. (2018) develop a model of care for OA [30]. Gay et al. (2015) carried out a systematic literature review on the education of patients on physical activity in OA care [31]. March et al. (2009) review three guidelines for OA [32]. Barton et al. (2021), Thorstensson et al. (2014) as well as Zywiel et al. (2021) perform a program evaluation [28, 33, 34]. MacKay et al. (2018) and Wallis et al. (2020) provide qualitative studies on factors influencing the referral to and participation in physical therapy in OA [35, 36]. Wallis et al. (2022) report a survey of patients using an OA program [37]. 

The included studies are from Australia [28–30, 32, 36, 37], Canada [34, 35] France [31], Sweden [28] and the United States of America [28] spanning from 2010 to 2022.

The OA-programs and models in the publications are Active with osteoarthritis (AktivA) – Norway [29], Amsterdam osteoarthritis cohort (AMSOA) – The Netherlands [27, 29], BOA Sweden [27]– [29], Enabling Self-management and Coping with Arthritic Pain using Exercise (ESCAPE-pain) – UK [29], GLA:D Australia [27–29, 36, 37], GLA:D Canada [29, 34], GLA:D China [29], GLA:D Denmark [29], GLA:D Switzerland [29], GLA:D New Zealand [29], Joint Academy - Sweden [29], Joint Health Program (JHP) – USA [29], Joint Implementation of Osteoarthritis guidelines in the West Midlands (JIGSAW) – Denmark, JIGSAW Netherlands [29], JIGSAW Norway [29], JIGSAW Portugal [29], JIGSAW United Kingdom [27, 29], Osteoarthritis Chronic Care Program Australia (OACCP) [27, 29], Osteoarthritis Healthy Weight For Life (OA-HWFL) Australia [27], The Joint Clinic – News Zealand [29] and Victorian Model of Care [30].

Additional file 1 presents metadata of the articles including information on the aim of the study, methods, samples, and participants as well as the OA program in focus.

### Results of the data extraction

**Fig. 4:**
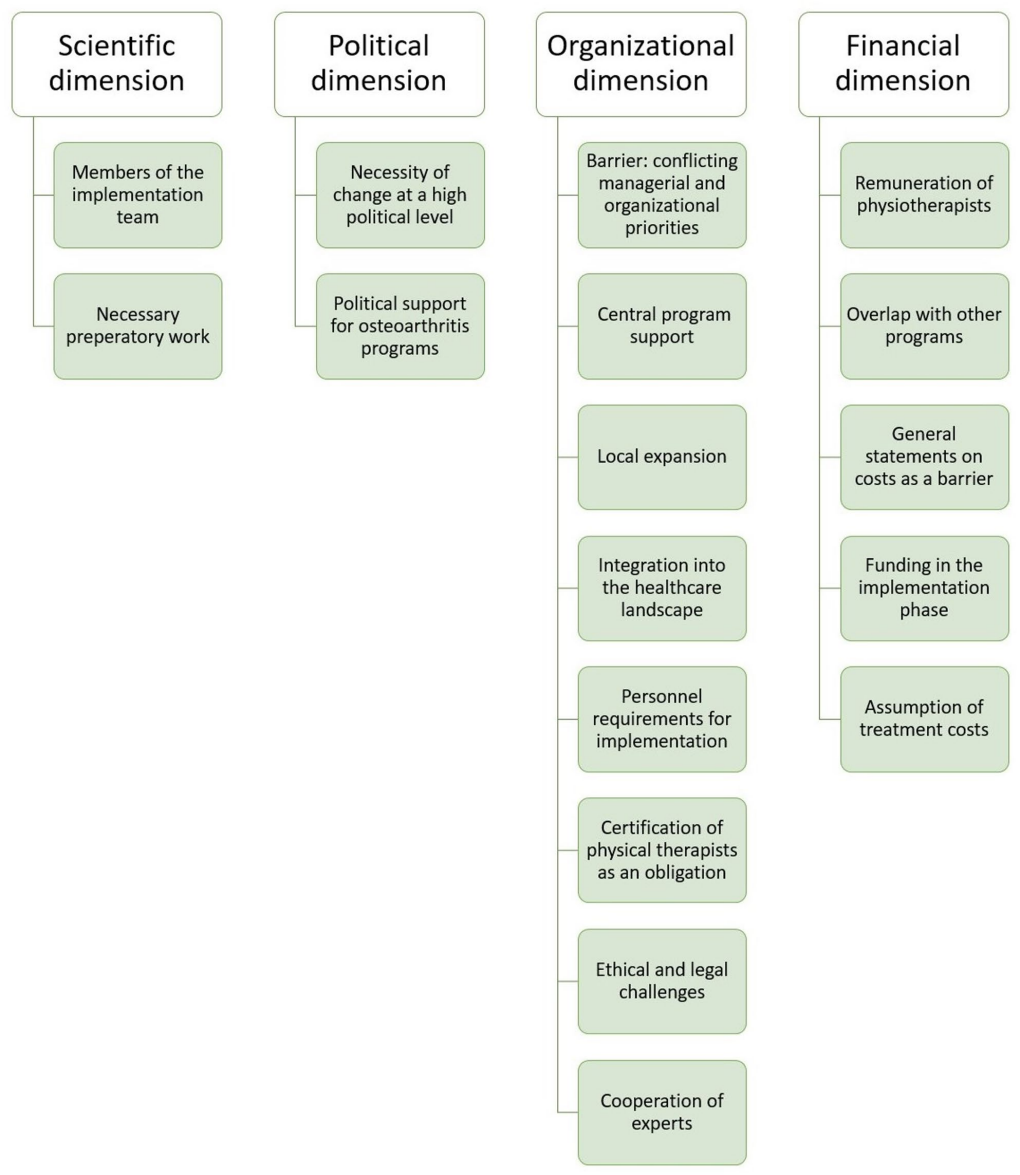
Categories and subcategories

We were able to identify scientific, political, organizational and policy concerns as most relevant factors for implementation, thus, resulting in the following superordinate categories, structuring the following presentation of the results: (1) Scientific dimensions, (2) Political dimensions, (3) Organizational dimensions and (4) Financial dimensions. The categories and subcategories can be found in Fig. 4 - Categories and subcategories.

The category “scientific dimension” comprises the subcategories “members of the implementation team” and “necessary preparatory work.” The category “political dimension” includes the subcategories “necessity of change at a high political level” and “political support for OA programs.” The category “organizational dimension” includes the subcategories “barrier: conflicting managerial and organizational priorities”, ”central program support”, ”local expansion”, ”integration into the healthcare landscape”, ”personnel requirements for implementation”, “certification of physical therapists as an obligation”, ”ethical and legal challenges”, and ”cooperation of experts.” The category “financial dimension” includes the subcategories “remuneration of physiotherapists”, “overlap with other programs”, “general statements on costs as a barrier”, “funding in the implementation phase”, and “assumption of treatment costs.”

### Scientific dimension

The scientific dimensions summarize all information that relates to the scientific support of the implementation. Scientific guidance plays an important role in the preparation and implementation process of treatment programs. It was found that the cooperation of the implementation team members with others is decisive: In their article Zywiel et al. (2021) report on a close coordination with the GLA:D^®^ Denmark team while implementing GLA:D^®^ in Canada. The collaboration allowed the authors to benefit from earlier scientific findings regarding the implementation of GLA:D^®^ in Denmark. This expertise contributed to the development of the necessary infrastructure, of the certification courses, the program support and the development of a quality and outcomes registry [34]. Zywiel at al. (2021) further use scientific methods to monitor the program fidelity of the physiotherapists and other musculoskeletal health care providers delivering the programs. They also looked for unanticipated barriers during the implementation process at different clinics in the sense of quality management. To this purpose, clinical leaders performed structured clinic reviews at ten different sites. Eight visits were carried out in person and remotely. The information gathered through the visits was incorporated into educational materials to improve program delivery and the implementation process at all sites [34].

### Political dimension

The political dimension describe political conditions relevant to implementation. The implementation of treatment programs should already begin at a (health) policy level, as the policy framework paves the way for successful implementation. For example, health policy and health planning can promote the implementation of treatment programs if those are included in health policy and corresponding financing agreements [30]. Similarly, health policy frameworks can be a constraining factor, as treatment programs depend on health policies that support their implementation [30]. March et al. further recognize that in order to implement treatment programs at the regional or national level, macro-level policies need to be in place that provide, for example, targets for funding, standards of care, and incentives for changes in care to healthcare professionals [32].

### Organizational dimension

The organizational dimension focus on all information addressing barriers and facilitating factors that have an impact on moving evidence-based non-invasive OA programs into standard care at the organizational level. This includes both personnel and geographic aspects. For example, Barton et al. report that central program support was a critical factor in the process of implementation. Safeguarding and the further development of the structured program as well as regular updates for the people involved, e.g. physiotherapists and project management, were mentioned as such factors [28]. The studies further identify a gradual implementation as a decisive factor. Zywiel et al. report that the program was first implemented in one city and then expanded statewide. The infrastructure was constantly scaled up, and the expansion was supported by program support (e.g. support from specific people in case of queries) [33]. Integrating the program into the supply landscape was a recurring topic in several publications. Wallis et al. report that broad availability in public and private healthcare settings at several locations is essential [37, 38] and can be achieved through better reimbursement [37]. Efficient referral processes into the programs are required [37]. The compatibility of the GLA:D^®^ program with the existing supply structure was further named as a relevant barrier in the course of integrating the program into the existing service structure. This was reinforced by sometimes contradictory managerial and organizational priorities [28]. Insufficient coordination and management were identified as one of the causes of inadequate OA care in the programs. Therefore, clinical pathways should be developed that incorporate core treatments [29]. Another organizational barrier in implementation is the limited authority of healthcare professionals in their regions to make decisions. This is challenging when new initiatives are being implemented in the region and healthcare professionals cannot act as stakeholders, due to limited decision-making authority [34].

At last, Zywiel et al. (2021) as well as Barton et al. (2021) point out an additional organizational challenge regarding certification, namely, to ensure sufficient staff for the implementation of the program in times of staff shortage [28, 34].

### Financial dimension

Finally, the financial dimension describes the effects of financing structures on the implementation process as well as the supply and demand of treatment programs. Funding of treatment programs has been addressed as a critical factor for implementation from several perspectives.

First, funding treatment programs can lead to fundamental barriers at the health system level. In this context, the lack of public funding for OA treatments in health systems has been listed as a barrier factor several times [28, 32, 35]. Zywiel et al. also identify funding as an issue when treatment programs have a real or perceived overlap with existing local or regional OA treatment programmes [34]. At the same time, the strict delineation of funding streams between disease management programs and health promotion or health prevention interventions is seen as a barrier to integrated care for OA patients [32]. Funding structures may further pose a barrier if unwillingness to invest in the health care system limits the effectiveness of interventions [31]. The implementation process itself is also dependent on available funding. Treatment programs rely on adequate funding to overcome initial implementation barriers and to ensure their successful implementation in practice [27, 28]. For example, in evaluating the implementation of GLA:D^®^ Australia, the lack of funding from health insurance was assessed as a constraining factor in implementation [28]. Wallis et al. reported that private health insurance rebates were important for program accessibility [37]. In this regard, it is also necessary to provide funding to the parties responsible for implementing treatment programmes [27, 37]. An example of such funding procedures is the implementation of GLA:D^®^ in Canada. The launch of the program was supported by a three-year grant from a foundation and the provision of infrastructure and staff by a university network. This initial support could be used by the program to build its own infrastructure for health service provision. Despite public funding, the program is still financed by self-payments and extended health benefits additionally [34]. The BOA project, for example, also received financial support from the National Social Insurance Agency and the Swedish government [33].

Another aspect is the impact of financing structures on health service provision. Briggs et al. identify flexible public and private financing models as a facilitating factor for health service provision [30]. Through a qualitative survey conducted as part of the GLA:D^®^ program in Australia, Barton et al. identify several requirements related to funding that should be met from the perspective of physical therapists to favor implementation [28]. The physiotherapists repeatedly mentioned the necessity of financing or partial financing, e.g., by health insurances as well as government subsidies, in order to financially secure the implementation and further provision of services.

Finally, financing structures also have an impact on access to and utilization of health care services by patients. Free access to treatment programs has been shown to increase patient adherence [31]. Nevertheless, the lack of cost reimbursement for patients and of public/private funding has been cited as a systematic barrier to program participation and an inhibiting factor to successful program implementation in several studies [28, 29].

In a qualitative study, physiotherapists state that the lack of funding results in patients not seeking the level of treatment they actually need [35]. In terms of perceived barriers to treatment program utilization, Wallis et al. and Barton et al. further note that cost is a barrier to participation [28, 38]. Factors such as government subsidies or discounts on private health insurance would influence a patient’s decisions about service utilization [38]. The physiotherapists continued to express concern in qualitative interviews about patients not seeking or delaying health care services due to financial barriers such as inadequate health insurance coverage or out-of-pocket payments [35].

Barton et al. and Zywiel et al. conclude that improved financing of treatment programs such as GLA:D^®^ is needed to increase access for patients [28, 34]. Additionally, the impact of different reimbursement models and their influence on service access should be further explored. Out-of-pocket payments continue to present a major barrier for patients that can result in disparities in access to health care services [34, 35].

## Discussion

### Interpretation and implications

The implementation of structured, non-invasive treatment programs for OA is progressing. To this end, treatment programs such as GLA:D^®^, which have already proven to be effective in several countries, are increasingly being adapted into further healthcare systems worldwide. Although a network has already developed around the established treatment programs, which promotes implementation internationally, there is little scientific evidence on the underlying implementation process. To clarify the implementation process and to identify promoting or hindering factors, the current state of research on evidence-based, non-invasive treatment programs for patients with OA of the knee and/or hip joint was examined. The results will be used as a basis for an expert-interview study on implementation hurdles regarding the implementation of analogue, digital and blended care as well as regional, national and international programs. As a result, four factors that seem to be of particular importance in the literature on implementing such programs were derived through the rapid review.

The **scientific dimension** revealed the relevance of collaborating with people who already gathered expertise in the implementation process in their countries as well as of preliminary work for implementation. The use of scientific methods and expertise to accompany the implementation process described previously showed that linking science and practice contributes to a structured implementation process of OA treatment programs. Previous research also identified the translation of evidence-based treatment methods derived from research into physiotherapy practice as challenging. A cooperation between academic staff and physiotherapists then facilitated the adoption and implementation of evidence-based interventions [39]. A synergy of practice and science thus contributes to maintaining the quality and effectiveness of evidence-based treatment programs in practice and ensuring structured integration into care.

Political conditions build the foundation for the implementation of treatment programs as shown in the section about the **political dimension**. Without regulations on financing structures or incentive systems for healthcare professionals, for example, regional or even national implementation of treatment programs are difficult to maintain in the long term. However, scientific proof of the effectiveness and added value of implementing a medical intervention, as in the case of treatment programs for OA, is not necessarily sufficient to initiate health policy action. In political decision-making, scientific evidence therefore competes with the values and interests of decision-making stakeholder groups, among others, but also with structural aspects such as the legal scope for action [40]. Part of the implementation strategy can therefore involve investigating how relevant stakeholders integrate scientific evidence into decision-making in health policy. This could involve using case studies to identify competing factors in decision-making in order to address them [40]. In this context, Bullock et al. argue that the integration of policy into implementation strategies is often only considered as a contextual factor instead of being a central component of the implementation strategy. The authors also point out that the term ‘policy’ is still used as a general term without further specification. With regard to the results of this review, this point of criticism can also be applied to the description of political factors in the included articles. The lack of consideration of the role of politics prevents precise allocation of responsibility and thus limits the possibility of incorporating health policy factors into implementation [41]. By deriving a set of recommendations, Crable et al. offer an approach for the requested specification of the health policy term [42]. The authors derive a set of recommendations to support researchers in making the role of health policy in the sustainable implementation of interventions transparent and suggest utilizing it for the implementation strategy [40]. For the implementation of OA treatment programs, this for example implies specifying the affecting policy regarding its intention, examining the development of the policy to understand its impact on the implementation or analyzing involved stakeholders.

The **organizational dimension** as third factor showed that the certification of physiotherapists for standardized treatment of OA of the knee and/or hip joint is decisive. The results of this rapid review are consistent with a study on the implementation of advanced musculoskeletal physiotherapy, that discovered that the knowledge, skills, availability, and motivation as well as experience of the physiotherapists had a large impact on the implementation [43]. Morris et al. strengthened this insight with their investigation on the sustainable implementation of extended-scope-of-practice in physiotherapy in Australia. There, physiotherapists undertake tasks exceeding the formally accepted practices. Providing a curriculum and accreditation was found to support physiotherapists in expanding their usual scope of practice, similar to a certification [44]. This supports the premise that strengthening professional authority and recognizing the knowledge of healthcare professionals is necessary for successful implementation.

A study where a clinical education framework was developed to support advanced musculoskeletal physiotherapy in Australia showed positive results while it was successfully implemented at six hospitals. While the results of this rapid review highlighted the importance of coordination and management as well as central program support, Harding et al. also noted that implementation requires a coherent and comprehensive pathway. The pathway was designed to adapt to local contexts, as the results in this present rapid review showed the need for a compatibility with existing structures [45]. Consisting of a competency-based training and assessment program, learning resources and a mentoring program it additionally considered the perspective of the patients as well as healthcare organizations and physiotherapists. This emphasizes the potentially positive results of using a multistep approach to develop a framework for implementation of physiotherapy. The process of translation of research into standard care can be supported by process models to plan the implementation in a structured way [46]. This is closely related to the investigated need for clinical pathways, efficient program referral processes as well as a central program support in this review.

The **financial dimension** was the most frequently addressed issue in the identified literature. This section has shown that a lack of financial resources can have a negative impact on treatment programs at all levels of care. Therefore, a lack of funding opportunities has an impact not only on the possibilities of program development and implementation in standard care but also on the effectiveness, supply, demand, and access to the treatment programs. It is therefore crucial to consider the origin of these financial barriers and to address them for a successful implementation process.

In addition to the four dimensions listed above, we identified the following associations in the literature: Proving the medical effectiveness of a treatment is considered mandatory before its implementation can even be addressed. By now, the economic evaluation of such treatment interventions is also becoming more relevant as their economic implications are an important source of information for decision-makers in healthcare and health politics. However, these economic evaluations often lack guidance on how decision-makers can use them to sustainably implement treatment interventions [47, 48]. Another aspect is that stakeholders are not only interested in the economic impact of the treatment intervention, but also in the additional costs associated with implementation as those are often not covered by health insurances for example. This can be a barrier since treatment programs will unlikely establish themselves in standard care if an initial implementation cannot be financed [47]. In this regard, the scoping review from Dropp et al. for example offers a tool against the lack of funding sources by explicitly presenting financing strategies for implementation in healthcare [49]. However, an economic assessment of the implementation costs does not only help decision-makers, but also disciplines scientists to develop pragmatic and feasible implementation strategies [47, 50].

### Strengths and limitations

The implementation process of evidence-based, non-invasive treatment programs for patients with OA of the knee- and/or hip joint has barely been examined in the scientific literature to date. Through a knowledge synthesis of the scientific literature, this review was able to identify relevant factors that need to be considered in the implementation of those treatment programs. One advantage of this work lies in the highly methodological approach applied in preparing the review. Despite conducting a rapid review, the search strategy was derived and documented in a structured manner in order to ensure a high degree of traceability of the methods. The search strategy was also advised by an expert for literature search from the library of the Ruhr-University Bochum and continuously assessed for plausibility by the team of authors. The qualitative content analysis and inductive derivation of the four thematic categories further ensured that all information from the scientific literature and relevant to the implementation was incorporated into the data extraction. The breadth of the search strategy ensured the inclusion of all relevant articles despite the diverse understanding of the word “implementation”.

However, there are some limitations to the results of this review. One difficulty in conducting this review was the lack of specific literature on implementation. When evaluating the results, it must therefore be considered that they represent an inductive derivation of implicit knowledge from literature. In addition, the perception of the term implementation varied across scientific literature. Within the publications addressing OA treatment programs. the transfer of programs into standard care, however, is consistently referred to as implementation. In order to maintain the balance between a comprehensive yet feasibly search in terms of a rapid review, we decided to use exclusively the search term “implementation”. This decision ensures the precision of the search but leads to the limitation that publications that address implementation in our sense but do not name it as such may have been omitted from our search. Considering these circumstances, the rapid review was a reasonable methodological approach to screen the big amount of literature resulting from the broader search strategy. By incorporating the programs ASReview Lab and Rayyan the authors were able to minimize this risk of relevant literature being omitted through semi-automatization and artificial intelligence (AI), which can be seen as another methodological strength. At the same time, however, it must be noted that the use of AI may lead to errors, too. Especially if the AI is trained with erroneous data. Our approach was selected based on weighing the advantages and disadvantages against each other.

Another limitation is that the literature search was conducted in September 2022 and could not be updated prior to publication. Due to the completion of the project, occupational changes among the authors and thereby a lack of resources, an update of the search was unfortunately not feasible for the authors. We acknowledge that this time gap may have resulted in the omission of more recent publications. Nevertheless, we are confident that the findings of this publication remain highly relevant as no review exists that specifically focuses on the implementation of non-invasive, evidence-based programs for OA of the knee and/or hip joint. Their results therefore provide a comprehensive synthesis of the relevant literature and may offer valuable insights for stakeholders involved in the implementation of such programs.

Future programs would benefit from updating this review, and we strongly encourage other research teams to build on and extend this work.

## Conclusion

The use and development of non-invasive evidence-based programs for patients with OA of the knee and/or hip joint which started in some Scandinavian countries is increasing. However, it remains a challenge to increase the availability of these programs in standard care through extensive implementation. Although a network which promotes implementation internationally has already developed some of the established treatment programs, there is limited scientific evidence on the underlying implementation process.

This review adds scientific evidence to the topic by identifying four factors of particular importance in the literature on implementing OA treatment programs: (1) The scientific dimensions, e.g. including the need for cooperation with other implementation-teams, (2) the political dimensions, e.g. including the provision of incentives for changes in care, (3) the organizational dimensions, e.g. including a need for central program support, and (4) the financial dimensions, e.g. including funding for evidence-based OA treatment programs.

## Supplementary Information

Below is the link to the electronic supplementary material.

Supplementary Material 1: Metadata table with further information on the articles included in the Rapid Review including information on authors, year of publication, title, country, aim of the study, methods, description of sample or participants, respective osteoarthritis program

## Data Availability

Data sharing is not applicable to this article as no datasets were generated or analyzed during the current study. Data extracted from the included studies can be found in the Metadata table. Data used for all analyses can be provided from the authors upon reasonable request. No analytic code was used. The search strategy can be found on https://www.cabidigitallibrary.org/action/doSearch?AllField=Theresa+Busse&SeriesKey=searchrxiv. In depth information on the included articles can be found in the metadata table in Additional file 1.
