# Supplementary material for: Promoting and inhibiting factors for the implementation of evidence-based non-invasive treatment programs for patients with knee- and/or hip-osteoarthritis: a rapid review
Source: BMC Health Serv Res. 2026 Mar 4;26:330. doi: 10.1186/s12913-026-14219-5 (PMC12964702; doi:10.1186/s12913-026-14219-5)
Supplement: Supplementary file 1 — Supplementary Material 1: Metadata table with further information on the articles included in the Rapid Review including information on authors, year of publication, title, country, aim of the study, methods, description of sample or participants, respective osteoarthritis program [file 12913_2026_14219_MOESM1_ESM.docx]

|  | **Authors** | **Title** | **Country** | **Aim of the study** | **Methods** | **Sample/**  **Participants** | **Osteoarthritis program** |
| --- | --- | --- | --- | --- | --- | --- | --- |
| 2010 | March L, Amatya B, Osborne RH, Brand C | Developing a minimum standard of care for treating people with osteoarthritis of the hip and knee | Australia | To provide implementation tools to support clinicians and customers to adopt and sustain implementation of a minimum standard of care, or a ‘core set’ of interventions, that should be offered to all patients with OA of the hip and/or knee | Review of three guidelines for the management of osteoarthritis and considering the evidence and potential for implementation | 3 published guidelines for management of osteoarthritis:   - OA Research Society International (OARSI) recommendations for the management of hip and knee OA, Part II: OARSI evidence-based, expert consensus guidelines. OARSI (2008) - OA: national clinical guideline for care and management in adults. National Institute of Clinical Excellence ((NICE) (2008) - Guideline for the non-surgical management of hip and knee OA, Royal Australian College of General Practice (RACGP) (2009) | / |
| 2014 | Thorstensson CA, Garellick G, Rystedt H, Dahlberg LE | Better Management of Patients with Osteoarthritis:  Development and Nationwide Implementation of an Evidence-Based Supported Osteoarthritis Self-Management Program | Sweden | To develop a supported osteoarthritis self-management program, delivered by trained physiotherapists, to facilitate patient and healthcare compliance | Evaluation of a program on osteoarthritis care at three- and twelve-month follow ups by data analysis | 20,200 consecutive patients in 320 different care centers in Sweden | Better Management of Patients with Osteoarthritis (BOA) |
|  | Allen KD, Choong PF, Davis AM, Dowsey MM, Dziedzic KS, Emery C, Hunter DJ, Losina E, Page AE, Roos EM, Skou ST, Thorstensson CA, van der Esch M, Whittaker JL | Osteoarthritis: Models for appropriate care across the disease continuum | USA | To describe the current evidence base for models of care (MoCs) across the spectrum of Osteoarthritis | Comparison of OA programs against recognized criteria for guideline implementation | 6 Featured programs in the Osteoarthritis Research Society International (OARSI) | - Osteoarthritis Chronic Care program (OACCP) – Australia - Better management of patients with osteoarthritis (BOA) – Sweden - Good Life with osteoarthritis in Denmark (GLA:D) – Australia - Osteoarthritis Healthy Weight For Life (OA HWFL) – Australia - Amsterdam osteoarthritis cohort (AMSOA) – The Netherlands - Joint Implementation of Osteoarthritis guidelines in the West Midlands (JIGSAW) – UK |
| 2016 | Gay C, Chabaud A, Guilley E, Coudeyre E | Educating patients about the benefits of physical activity and exercise for their hip and knee osteoarthritis. Systematic literature review | France | To highlight the role of patient education about physical activity and exercise in the treatment of hip and knee osteoarthritis | Systematic literature review (Cochrane Library, PubMed, Wiley Online Library) | 13 randomized controlled trials and 8 recommendations were reviewed (1b level of evidence) | / |
| 2018 | Briggs AM, Page CJ, Shaw BR, Bendrups A, Philip K, Cary B, Choong PF | A Model of Care for Osteoarthritis of the Hip and Knee: Development of a System-Wide Plan for the Health Sector in Victoria, Australia | Australia | To develop a model of care for osteoarthritis and to present lessons learned | Development of the model of care by using a best-practice framework including a survey and a workshop | - 75 survey respondents - Workshop with External Expert Advisory Committee of 25 clinical/service champions - 43 respondents to survey on full draft of Model of Care | Victorian Model of Care (development by following best-practice framework, best evidence and iterative cross-sector consultation) |
| 2018 | MacKay C, Hawker GA, Jaglal SB | Qualitative study exploring the factors influencing physical therapy management of early knee osteoarthritis in Canada | Canada | To identify the perceived barriers and facilitators to managing clients with early knee OA and to identify the contextual factors affecting implementation of care by physical therapists | Qualitative study including an inductive and thematic analysis of in-depth semi-structured interviews with 33 physical therapists | Purposive sample of 33 physical therapists from private practice and publicly funded settings who managed clients with knee symptoms and/or diagnosed knee OA  in community/outpatient settings in three provinces in Canada (Ontario, Alberta, British Columbia) | / |
| 2020 | Bowden JL, Hunter DJ, Deveza LA, Duong V, Dziedzic KS, Allen KD, Chan PK, Eyles JP | Core and adjunctive interventions for osteoarthritis: efficacy and models for implementation | Australia | To discuss core priority treatments and evidence-based physical or psychological adjunctive therapies and combined therapies for osteoarthritis and to give examples of international osteoarthritis treatment options | Discussion of and guidelines, including examples of available programs, models of care and resources that are available to support these recommendations | - Recommendations and guidelines from international context and leading organizations - 10 examples of international programs, models of care and resources | - Osteoarthritis Chronic Care program (OACCP) – Australia - Good Life with osteoarthritis in Denmark (GLA:D®) – Denmark, Canada, Australia, China, Switzerland, New Zealand - Better management of patients with osteoarthritis (BOA) – Sweden - Joint Academy – Sweden - Active with osteoarthritis (AktivA) – Norway - Enabling Self-management and Coping with Arthritic Pain using Exercise (ESCAPE-pain) – UK - Amsterdam osteoarthritis cohort (AMSOA) – The Netherlands - Joint Implementation of guidelines for osteoarthritis guidelines in western Europe (JIGSAW) - UK, Denmark, Netherlands, Norway, Portugal - Joint Health Program (JHP) - USA - The Joint Clinic - New Zealand |
| 2020 | Wallis JA, Acherman IN, Brusco NK, Kemp JL, Sherwood J, Young K, Jennings S, Trivett A, Barton CJ | Barriers and enablers to uptake of a contemporary guideline-based management program for hip and knee osteoarthritis: A qualitative study | Australia | To explore barriers and enablers for referral to, and participation in, a guideline-based osteoarthritis management program – Good Life with osteoArthritis in Denmark (GLA:D® Australia) and to develop a set of recommendations for optimization | Qualitative analysis of semi-structured interviews with patients with osteoarthritis and medical professionals using the theoretical domains framework and development of recommendations | - 20 patients with hip or knee osteoarthritis - 15 medical professionals (5 general practitioners, 4 rheumatologists, 6 orthopedic surgeons) | - Good Life with osteoArthritis in Denmark (GLA:D® Australia) |
| 2021 | Barton CJ, Kemp JL, Roos EM, Skou ST, Dundules K, Pazzinatto MF, Francis M, Lannin NA, Wallis JA, Crossley KM, | Program evaluation of GLA:D® Australia: Physiotherapist training outcomes and effectiveness of implementation for people with knee osteoarthritis | Australia | To evaluate the implementation of Good Life with osteoArthritis from Denmark (GLA:D®) for knee osteoarthritis in Australia using the RE-AIM QuEST (Reach, Effectiveness, Adoption, Implementation, Maintenance Qualitative Evaluation for Systematic Translation) framework | Program evaluation guided by RE-AIM QuEST framework based on Cohort study of the implementation of GLA:D® Australia | - Survey data from 1064 physiotherapists - Baseline data from 1945 patients, of which 1044 completed a 3-month follow up and 927 completed a 12-month follow up | Good Life with Osteoarthritis in Denmark Australia (GLA:D® Australia) |
| 2021 | Zywiel MG, Ellis K, Veillette CJH, Skou ST, McGlasson R | Implementation of the Good Life with osteoArthritis in Denmark (GLA:D) Program across Canada for the Management of Hip and Knee Osteoarthritis | Canada | - To present the adaptation of GLA:D in Canada - To highlight the key program and participant outcomes after four years of implementation - To discuss the benefits of implementing a national standardized program, available in both the public and private sectors, for individuals with hip and knee osteoarthritis | Program evaluation using data from the implementation of GLA:D® Canada (2016-2020) | Data collected over a 4-year period (2016-2020) concerning the implementation, adaption, and outcomes of GLA:D® Canada | Good Life with osteoArthritis in Denmark (GLA:D® Canada) |
| 2022 | Wallis JA, Barton CJ, Ackerman IN, Sherwood J, Kemp JL, Young K, Jennings S, Trivett A, Brusco NK | A survey of patient and medical professional perspectives on implementing osteoarthritis management programs for hip and knee osteoarthritis | Australia | - To investigate and compare patients’ and medical professionals’ views around hip and knee osteoarthritis management and factors impacting implementation of an osteoarthritis management program - To discover the convergence of the findings between patients and medical professionals | - Descriptive analysis of data from an online survey as part of a mixed-methods program of research for patients with hip or knee osteoarthritis and medical professionals routinely involved in management of osteoarthritis (chi squared tests) - Triangulation with previously reported qualitative data | - 53 patients - 32 medical professionals | Good Life with osteoArthritis in Denmark (GLA:D®) Australia |
